# Effects of Seasonal Operation on the Quality of Water Produced by Public-Supply Wells

**DOI:** 10.1111/gwat.12174

**Published:** 2014-03-04

**Authors:** Laura M Bexfield, Bryant C Jurgens

**Affiliations:** 2 U.S. Geological Survey, Placer Hall6000 J St., Sacramento, CA 95819

## Abstract

Seasonal variability in groundwater pumping is common in many places, but resulting effects of seasonal pumping stress on the quality of water produced by public-supply wells are not thoroughly understood. Analysis of historical water-quality samples from public-supply wells completed in deep basin-fill aquifers in Modesto, California (134 wells) and Albuquerque, New Mexico (95 wells) indicates that several wells have seasonal variability in concentrations of contaminants of concern. In Modesto, supply wells are more likely to produce younger groundwater with higher nitrate and uranium concentrations during the summer (high) pumping season than during the winter (low) pumping season. In Albuquerque, supply wells are more likely to produce older groundwater with higher arsenic concentrations during the winter pumping season than during the summer pumping season. Seasonal variability in contaminant concentrations in Modesto is influenced primarily by effects of summer pumping on vertical hydraulic gradients that drive migration of shallow groundwater through the aquifer to supply wells. Variability in Albuquerque is influenced primarily by the period of time that a supply well is idle, allowing its wellbore to act as a conduit for vertical groundwater flow and contaminant migration. However, both processes are observed in each study area. Similar findings would appear to be likely in other alluvial basins with stratified water quality and substantial vertical head gradients. Results suggest that even in aquifers dominated by old groundwater, changes to seasonal pumping patterns and/or to depth of well completion can help reduce vulnerability to selected contaminants of either natural or anthropogenic origin.

## Introduction

A detailed understanding of factors affecting contaminant occurrence and concentrations in water from public-supply wells in various hydrogeologic settings is critical to the effective allocation of limited resources for groundwater protection and resource management. Although the vulnerability of water from supply wells to natural and anthropogenic contaminants is known to be dependent on characteristics of pumping stresses on the groundwater system (Reilly and Pollock 1993; Focazio et al. 2002), the effects of seasonal variability in pumping stresses on water quality have not been widely studied. In many aquifers, variations in water quality may be linked to seasonal changes in water demand that require wells used for irrigation and public supply to be pumped more often and for longer periods of time during the summer than during the winter. This transience in well operation can cause significant variability in hydrologic conditions, particularly with respect to horizontal and vertical hydraulic gradients that drive transport of contaminants through the groundwater system. Changes in the direction or magnitude of hydraulic gradients caused by seasonal pumping can be particularly pronounced in areas where surface water supplies are insufficient to meet summer water demand, such as in the semiarid to arid southwestern United States. Additional knowledge of these effects might allow optimization of well operations to minimize vulnerability of water from some supply wells to contaminants of greatest concern, and could improve the ability to detect multi-year water-quality trends that are masked by seasonal variability.

Multiple investigations have sought to characterize sources of contaminants to public-supply wells by studying observations of groundwater chemistry and age (Aeschbach-Hertig et al. 1998; Manning et al. 2005; Katz et al. 2007; Jurgens et al. 2008; Landon et al. 2008; McMahon et al. 2008; Plummer et al. 2008; Brown et al. 2009; Hinkle et al. 2009; Katz et al. 2009; Landon et al. 2010b; Ayotte et al. 2011; Musgrove et al. 2011; Bexfield et al. 2012; Eberts et al. 2012); however, few studies appear to have used these observational data to explore whether seasonal variability in well operations can have important effects on water quality. Seasonal to multi-year variability has been described for public-supply wells in karst systems dominated by relatively young water (commonly less than about 50 years) (Katz et al. 2007; Musgrove et al. 2011), where the quality of water from supply wells could be expected to respond to even short-term changes in hydrologic conditions. Results of these studies, though, are not clearly applicable outside of karst systems and do not distinguish the potential effects of supply well pumping from the effects of seasonal changes in recharge.

Investigations conducted by the U.S. Geological Survey (USGS) of individual public-supply wells in Modesto, California in 2003 to 2005 (Jurgens et al. 2008) and Albuquerque, New Mexico in 2007 to 2009 (Bexfield et al. 2012) found seasonal patterns in contaminant concentrations, even though the wells were completed in deep basin-fill aquifers with relatively long travel times (averaging hundreds or thousands of years). Seasonal changes in recharge would not be expected to affect supply well water quality in such aquifer systems. These investigations, which were conducted as part of the National Water-Quality Assessment program's study of the Transport of Anthropogenic and Natural Contaminants (TANC) to public-supply wells (Eberts et al. 2013), used data on groundwater chemistry, hydraulic heads, and wellbore flow to demonstrate that seasonal pumping patterns and associated transience in hydrologic conditions in the vicinity of the two studied supply wells led to the observed patterns in water quality.

The purpose of this paper is to describe the regional occurrence of seasonal patterns in contaminant concentrations in groundwater produced by public-supply wells in the two alluvial basins where the Modesto and Albuquerque TANC studies were conducted and to explain the causes of the seasonal patterns observed. Hydraulic-head and wellbore-flow observations are used to identify the processes by which temporal changes in hydrologic conditions and flow dynamics influence seasonal patterns in contaminant concentrations. These processes are then examined for transferability to other alluvial basins. Results are also used to identify ways in which supply wells affected by such hydrologic processes could be constructed and/or operated to reduce their vulnerability to selected contaminants of concern.

## Study Area Descriptions

### Modesto, California, USA

**Figure 1:**
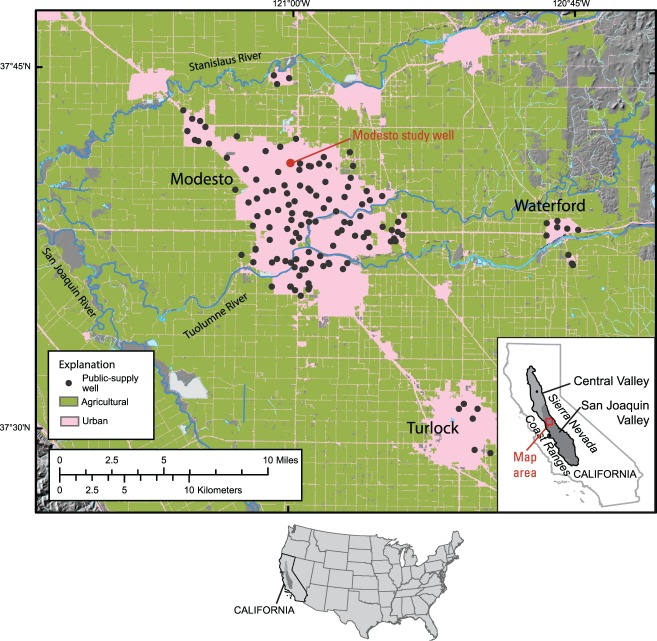
Physiographic features, land use, and locations of public-supply wells analyzed for seasonal variability in water quality near Modesto, San Joaquin Valley, California.

Modesto is located in the semiarid San Joaquin Valley, which occupies the southern two-thirds of the Central Valley of California (Figure 1). The Central Valley is a large, asymmetric structural trough filled with marine and continental sediments up to 10,000 m thick (Page 1986; Gronberg et al. 1998). The San Joaquin Valley has a level depression more than 400 km long and 30 to 90 km wide that is bounded on the east by the Sierra Nevada and on the west by the Coast Ranges. The alluvial fill of the basin used for drinking water is composed primarily of unconsolidated fluvial deposits of sand, gravel, clay, and silt, primarily of Pleistocene and Holocene age (Burow et al. 2004). The aquifer system can be generally described as an unconfined to semi-confined aquifer, underlain by a confined aquifer where a regional lacustrine clay unit commonly referred to as the Corcoran Clay occurs (Williamson et al. 1989; Bertoldi et al. 1991). Depth to water varies from more than 20 m near the Valley margins in the east to less than 3 m near the axial trough in the center of the basin. Recharge to the aquifer is primarily from agricultural irrigation, seepage from streams and rivers entering the valley near the mountain fronts and, to a lesser extent, from precipitation. Pumping is the primary form of groundwater discharge. Groundwater not captured by pumping wells discharges to the San Joaquin River, which drains the northern part of the San Joaquin Valley through San Francisco Bay, or to major tributaries.

About 65% of the study area is planted in irrigated crops. In Stanislaus County, including Modesto, the estimated population was more than 500,000 people in 2005 (California Department of Finance, 2006). Urban water demand is met by surface water and groundwater supplies. Before 1995, drinking water for the City of Modesto was supplied by groundwater only. In 1994, a surface water treatment plant was completed, which now provides about half of Modesto's municipal and industrial water supply.

Public-supply wells in the vicinity of Modesto generally range in depth from about 18 m to over 120 m and commonly have screened intervals exceeding 20 m in length. Previous investigations have shown that groundwater produced by these wells can be entirely young (post-1950) groundwater, entirely old (pre-1950) groundwater, or a mixture of both young and old (Wright et al. 2004; Landon et al. 2010a). The primary groundwater contaminants that may pose a significant threat to the long-term sustainability of groundwater in the Modesto area are nitrate and uranium. Concentrations above the U.S. Environmental Protection Agency (USEPA) maximum contaminant level (MCL) of 45 mg/L for nitrate (as NO_3_) (U.S. Environmental Protection Agency 2010a) and the California MCL of 20 pCi/L for uranium (California Department of Public Health 2007) were recently found in about 5 and 6%, respectively, of public-supply wells in the Central-Eastside San Joaquin Valley (Landon et al. 2010a). Elevated nitrate concentrations in groundwater in the eastern San Joaquin Valley are derived mainly from the application of nitrogen-based fertilizers for agricultural purposes (Burow et al. 2008b). Uranium, on the other hand, occurs naturally in the sediment and soil. It is normally bound to the sediment, but agricultural practices favor the formation of high-bicarbonate water that infiltrates the soil, mobilizing uranium from the sediment and into solution in water that eventually reaches the water table as recharge (Jurgens et al. 2010). Thus, nitrate and uranium typically are associated with shallow, young groundwater that has been affected by agricultural activities (Wright et al. 2004; Burow et al. 2008a, 2008b; Jurgens et al. 2008, 2010; Landon et al. 2010a); concentrations are lower in older, deeper groundwater.

### Albuquerque, New Mexico, USA

**Figure 2:**
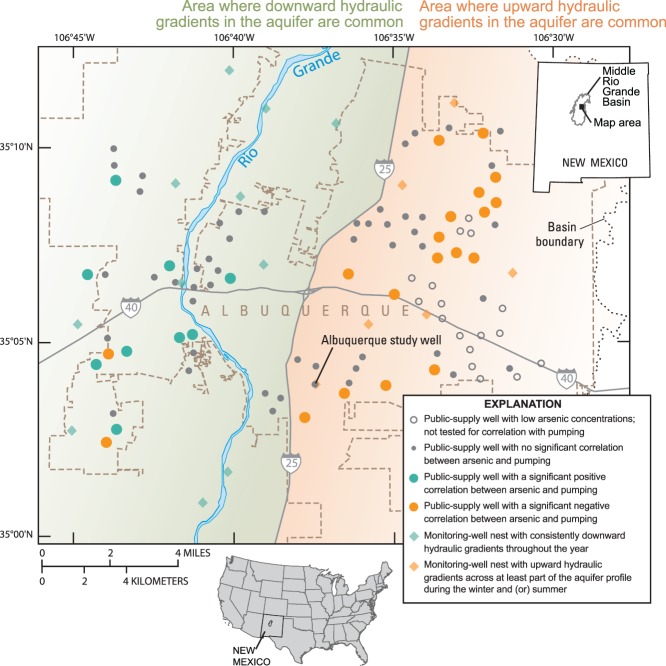
Locations of public-supply wells analyzed for seasonal variability in water quality near Albuquerque, Middle Rio Grande Basin, New Mexico, indicating relations between arsenic and pumping. Also shown are locations of monitoring-well nests with associated directions of vertical hydraulic gradients. Public-supply wells with a significant positive correlation between arsenic and pumping are located in areas with primarily downward hydraulic gradients throughout the year, whereas wells with a significant negative correlation are located mainly in areas with upward hydraulic gradients across at least part of the aquifer profile during the winter and (or) summer.

Albuquerque is located in the semiarid Middle Rio Grande Basin of central New Mexico (Figure 2). The 7922-km^2^ Middle Rio Grande Basin trends generally north to south and contains alluvial fill up to about 4500 m thick. The basin is bounded primarily by mountains on the north and east, and by several smaller uplifts, a fault zone, and an adjacent structural basin on the west. The alluvial fill of the basin is composed principally of the unconsolidated to moderately consolidated Santa Fe Group deposits of late Oligocene to middle Pleistocene age that were deposited in fluvial, lacustrine, or piedmont-slope environments. These deposits, in combination with hydraulically connected overlying basin-fill deposits of Pleistocene to Holocene age, form the Santa Fe Group aquifer system (Thorn et al. 1993). Conditions within the aquifer system are generally unconfined, but they are semi-confined at depth. Depths to water in the general vicinity of Albuquerque range from about 1 m to more than 200 m, with generally smaller depths to water near the Rio Grande.

Recharge to the aquifer system of the Middle Rio Grande Basin is primarily through seepage from the Rio Grande and associated irrigation canals, although mountain-front recharge, subsurface groundwater inflow, and urban sources including leakage from water distribution systems also contribute water to the aquifer (Bexfield et al. 2011a). Groundwater discharges from the aquifer system through agricultural drains, groundwater withdrawals for public supply, and riparian evapotranspiration. Until the Albuquerque Bernalillo County Water Utility Authority (ABCWUA) began in 2008 to meet some water demand with surface water diverted from the Rio Grande, essentially all drinking water for residents of the Albuquerque metropolitan area (population 713,000 in 2000; U.S. Census Bureau 2001) was supplied by groundwater withdrawals. Public-supply wells in the vicinity of Albuquerque generally range in depth from about 150 m to nearly 550 m and commonly have screened intervals exceeding 150 m in length. Groundwater produced by these wells is dominantly thousands of years old, but can include a small fraction that recharged within the past 50 years (Plummer et al. 2004a, 2004b; Bexfield et al. 2012).

Elevated concentrations of arsenic in groundwater across large areas of the Middle Rio Grande Basin pose a challenge for the use of groundwater for drinking water supply, particularly since the lowering of the MCL for arsenic from 50 to 10 µg/L (effective in 2006). Utilities have installed wellhead treatment systems at public-supply wells producing water with elevated arsenic concentrations and/or have blended groundwater from these wells with groundwater from other wells or with surface water. Because the ABCWUA uses groundwater to supplement its primary surface water supply during drought and during times of peak demand, the concentration of arsenic in water from individual wells continues to affect management of the municipal supply. Investigations by Bexfield and Plummer (2003) and by Plummer et al. (2004a) found that concentrations of arsenic above the USEPA MCL of 10 µg/L (U.S. Environmental Protection Agency 2010a) in the area typically are associated with older and/or deeper groundwater. These investigations concluded that sources of elevated concentrations of arsenic include inflow of high-arsenic groundwater related to silicic volcanism in mountains north of the basin, and upwelling of mineralized water of deep origin along major structural features across the basin. Contaminants of anthropogenic origin also are of concern. In particular, VOCs have been detected in public-supply wells in the Albuquerque area, generally at concentrations far below USEPA MCLs (Plummer et al. 2008; Bexfield et al. 2012), but occasionally at concentrations that required closure of a supply well located near a contamination site (U.S. Environmental Protection Agency 2010b, 2010c).

## Methods

### Data Sources

Regional-scale datasets of historical water quality were obtained from the City of Modesto for 138 wells that it has used for public supply (Figure 1) and ABCWUA for 95 wells that it has used for public supply (Figure 2). The historical data were collected from the Modesto wells between 1984 and 2008 and from the Albuquerque wells between 1988 and 2005. Unfiltered samples were generally collected by the utilities on an annual or semi-annual basis from the wellhead of each operational supply well, prior to any treatment and after some minimum purge time (documented to be 2 h for the ABCWUA). Sample results therefore represent the quality of the raw groundwater resource and not necessarily of the finished drinking water delivered to consumers, which has been treated and/or blended as needed to ensure compliance with drinking water standards. Analytes included field measurements, major and trace elements, and nutrients. Analysis of samples collected from the City of Modesto wells was conducted by various certified State-approved laboratories and reported to the California Department of Public Health. Analysis of samples collected from ABCWUA wells was conducted by the utility's water-quality laboratory. Table 1 summarizes the available historical water-quality data and corresponding supply well characteristics in each study area.

**Table 1:** Summary of Historical Water-Quality Databases Used for Investigation in the Modesto and Albuquerque Study Areas

StudyArea	Source/Collecting Agency	Total Number of Wells	Range of Well Depth (mbls)	Range of Length of Screened Interval (m)	Range of Depth to Water (mbls)	Years of Data Collection	Typical Frequency of Data Collection	Types of Available Water-Quality Data	Availability of Pumping Data
Modesto, CA	City of Modesto	138	18-125	0.3–91.5	<3–20	1984–2008	Annual to quarterly	Major, minor, and trace elements; Nutrients Field measurements	Monthly for aggregated wells
Albuquerque, NM	Albuquerque Bernalillo County Water Utility Authority	95	152–544	43–309	3.4–234	1988–2005	Annual to biannual	Major, minor, and trace elements;Nutrients Field measurements	Monthly for individual wells

**Figure 3:**
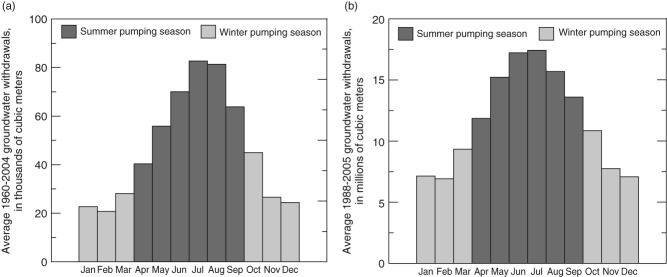
Average monthly groundwater withdrawals for all public-supply wells operated by (a) the City of Modesto, 1960 to 2004 and (b) the Albuquerque Bernalillo County Water Utility Authority, 1988 to 2005. Withdrawals typically are greater during months in the summer pumping season than during months in the winter pumping season.

Monthly groundwater withdrawals were available for individual wells operated by the ABCWUA during 1988 to 2005 and for the entire group of wells (but not individual wells) operated by the City of Modesto during 1960 to 2004. These data indicated that withdrawals typically were substantially greater during the summer pumping season (April through September) than during the winter pumping season (October through March; Figure 3). Despite slight differences in seasonal pumping patterns, the same pumping seasons were assigned to both study areas for consistent seasonal analysis.

The Modesto study well (MSW; Figure 1) investigated by the TANC study is relatively deep for supply wells in the Modesto area, but produces water containing concentrations of nitrate and uranium occasionally approaching or above the MCL, respectively, along with low concentrations of pesticides and volatile organic compounds (VOCs). The MSW is cased to 114.9 meters below land surface (mbls), with a screened interval extending from 27.7 to 111.6 mbls; the well has a water level of about 10.2 mbls, and produces water at an average rate of about 6.1 m^3^/min (1600 gpm) (Jurgens et al. 2008). The Albuquerque study well (ASW; Figure 2) is constructed in a typical manner for supply wells in the Albuquerque area, and produces water containing concentrations of arsenic occasionally above the MCL, along with low concentrations of VOCs. The ASW is cased to 363 mbls, with a screened interval extending from 107 to 359 mbls; the well has a water level of about 75.8 mbls, and produces water at an average rate of about 11.7 m^3^/min (3080 gpm) (Bexfield et al. 2012). The MSW and ASW are each screened across a single geologic unit composed of unconsolidated fluvial deposits of alternating sand, gravel, clay, and silt, without any consistent trend in hydraulic conductivity with depth. Testing conducted on the MSW and ASW during the TANC study (including chemical and flow testing and camera surveys) did not indicate evidence of damage to seals or corrosion that could affect flow to or within the wells; however, mineral deposits observed in the MSW might decrease the ability of perforations above 39.6 mbls to transmit water from the aquifer (Jurgens et al. 2008).

For the TANC study, the MSW was sampled in 2003 to 2004 and the ASW was sampled in 2007 to 2009 for a large suite of analytes, including age tracers. Samples were collected prior to any treatment and after at least three casing volumes of water had been purged from the wells. Samples collected for contaminants discussed in this paper (nitrate, uranium, and arsenic) were filtered with a 0.45-µm capsule filter. Details of the collection, processing, and laboratory analysis of samples collected through the TANC study are provided for the MSW by Jurgens et al. (2008) and for the ASW by Bexfield et al. (2012).

Available hydraulic-head data for the Modesto and Albuquerque study areas were used to characterize vertical gradients and their response to seasonal pumping. Nests of monitoring wells drilled for the TANC study in the vicinity of the MSW (seven nests) and ASW (five nests), which included one nest located within about 33 m of each study well, were instrumented with continuous water-level monitors. Regionally across the Albuquerque area, continuous hydraulic-head data were available for 17 nests of monitoring wells that include two or more wells screened at least 100 m apart, generally across the water table, near the middle of the production zone, and near the bottom of the production zone. In addition, flow of water within the wellbore of the ASW was observed using an electromagnetic flowmeter under pumping and nonpumping conditions.

### Statistical Methods

Nonparametric, rank-based methods of statistical analysis were used to investigate patterns in historical concentrations of selected contaminants of concern from the public-supply wells because the data were not normally distributed and censored values were present for some analytes. Censoring levels did not change through time for any of the analytes examined in this study. For statistical testing, censored values were assigned a single value less than the lowest quantified concentration for that analyte. The Mann-Whitney test, which tests for differences in median values between two groups of independent (unpaired) observations, was used to examine whether contaminant concentrations from an individual supply well were significantly different between the summer and winter pumping seasons. The signed-rank test was not feasible for individual wells because most wells did not have matching pairs of samples for every year, but it was used to compare concentrations across multiple wells. Kendall's tau (τ), which measures the strength of the monotonic relation between variables, was used to investigate correlations between concentrations and pumping rates. The significance level used for all tests was 0.05.

For individual ABCWUA wells, contaminant concentrations were tested for correlation with monthly pumping volume. Given the resolution of the pumping data, pumping during the 1-month period prior to collection of an individual sample was assumed to have the greatest influence on the water quality observed for that sample. Therefore, monthly pumping volumes were assigned to the water-quality data as follows: if the sample was collected during the first 10 d of the month, the previous month's pumping total was assigned to the sample; if the sample was collected during the second 10 d of the month, the average of the previous and current months' pumping totals was assigned; and if the sample was collected during the last 8 to 11 d of the month, the current month's pumping total was assigned.

## Results and Discussion

### Seasonal Trends in Supply Well Water Quality in Modesto

Of the 138 Modesto supply wells with a nitrate sample and 135 wells with a uranium sample, 31 wells (22%) had at least one nitrate concentration above the USEPA MCL of 45 mg/L (as NO_3_) and 43 wells (32%) had at least one uranium sample above the CA MCL of 20 pCi/L. However, median concentrations measured in raw samples from all supply wells were 18 mg/L for nitrate and 5 pCi/L for uranium (less than one half of the corresponding MCL).

Most public-supply wells in the Modesto area produce groundwater with seasonal differences in nitrate and/or uranium concentrations, and concentrations for samples collected during the summer pumping season (April through September) generally are higher than concentrations for samples collected during the winter pumping season (October through March). Of 134 wells with nitrate data for each season, 122 (91%) had seasonal differences in median concentrations of at least 0.2 mg/L and 59 (44%) had seasonal differences of at least 3 mg/L. Of 117 wells with uranium data for each pumping season, 103 (88%) had seasonal differences in median concentrations of at least 0.1 pCi/L and 40 (34%) had seasonal differences of at least 3 pCi/L. The median nitrate concentration for all wells was 19 mg/L (as NO_3_) during the summer and 17 mg/L during the winter (signed-rank test, p = 0.002). Similarly, the median uranium concentration for all wells was 5.7 pCi/L during the summer and 5.0 pCi/L during the winter (signed-rank test, p = 0.001).

**Figure 4:**
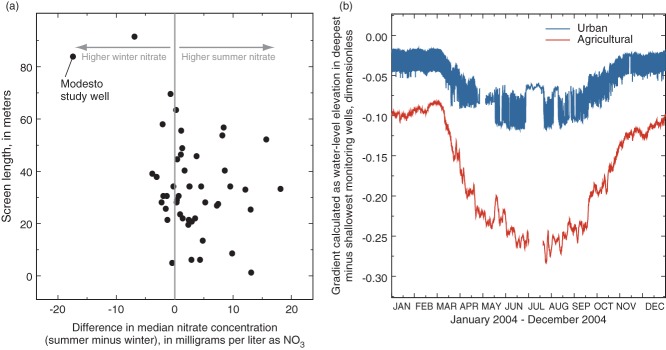
(a) In the Modesto dataset, substantially more wells had higher median concentrations of nitrate during the summer pumping season than during the winter pumping season. The Modesto study well had the opposite seasonal pattern. (b) Vertical hydraulic gradients in representative monitoring-well nests in the Modesto area are downward throughout the year (negative values indicate downward gradients). These gradients are greatest during the summer as a result of increased pumping from depth for irrigation and public supply, likely drawing more shallow groundwater with elevated nitrate concentrations to depths where it can be captured by most supply wells.

Mann-Whitney tests conducted on 66 individual wells with more than 10 nitrate samples in each seasonal category indicated that 55% (36) had significantly higher concentrations for samples collected during the summer, whereas 17% (11) had significantly higher winter concentrations. For the 36 wells with more than 10 uranium samples in each seasonal category, Mann-Whitney tests indicated that 22% (8) had significantly higher summer concentrations and 6% (2) had significantly higher winter concentrations. Because past studies have shown that nitrate and uranium are highest in shallow groundwater that has been affected by agricultural activities (Wright et al. 2004; Burow et al. 2008a, 2008b; Jurgens et al. 2008, 2010; Landon et al. 2010a), the prevalence of higher concentrations of these contaminants in summer samples (Figure 4a for nitrate) implies that a greater contribution of young, shallow groundwater is captured by most supply wells during the summer as compared to the winter.

**Figure 5:**
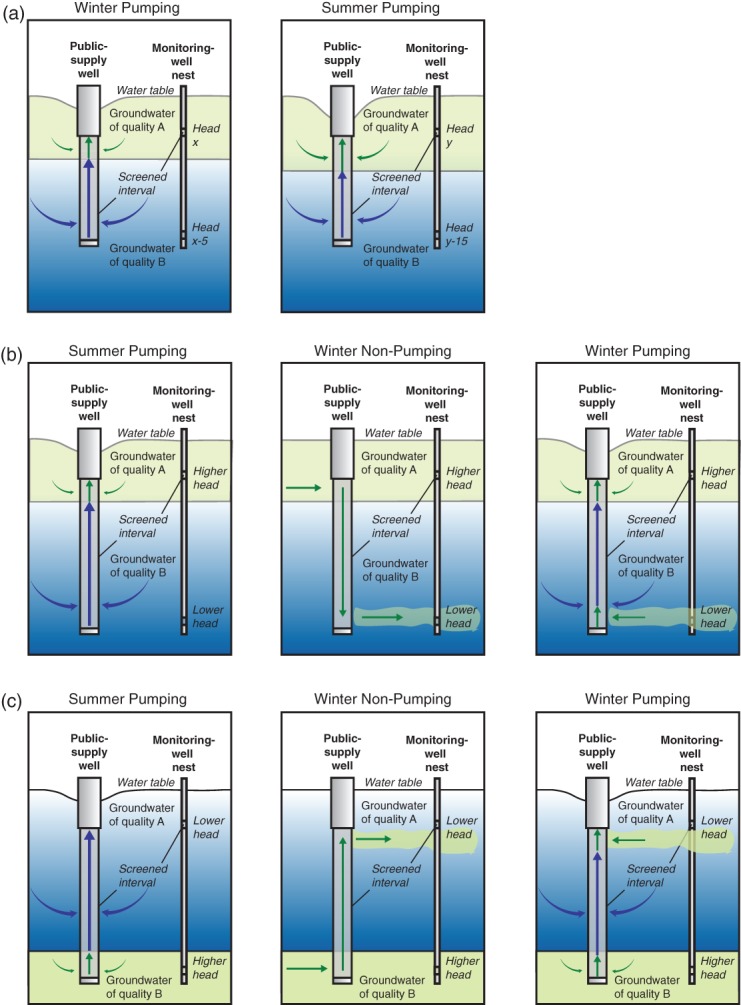
Conceptual models of hydrologic processes influencing seasonal variability in the quality of water produced by public-supply wells in the Modesto and Albuquerque study areas, and likely in other alluvial basins with stratified water quality and substantial vertical head gradients. (a) Conceptual model where the dominant process is enhanced downward migration of shallow contaminated groundwater through the aquifer to the screened intervals of supply wells as a result of increased regional pumping during the summer. (b) Conceptual model where the dominant process is downward flow of shallow contaminated groundwater through wellbores of idle supply wells in the winter to greater depths where the water resides until pumping resumes. (c) Conceptual model where the dominant process is upward flow of deep contaminated groundwater through wellbores of idle supply wells in the winter to shallower depths where the water resides until pumping resumes.

Hydraulic heads recorded in monitoring wells located in northeast Modesto (Jurgens et al. 2008) indicate that vertical hydraulic gradients generally are downward throughout the year under both pumping and ambient (nonpumping) conditions (Figure 4b). The gradient typically is greatest between the shallow and intermediate parts of the aquifer, where semi-confined conditions become pervasive. During the summer, public-supply wells and agricultural wells tend to be pumped longer and more frequently. In addition to groundwater withdrawals, application of irrigation water for crops in agricultural areas and for landscapes in urban areas causes vertical hydraulic gradients between the shallow and deeper zones to reach a maximum during the summer when water demand is high (Phillips et al. 2007; Burow et al. 2008a; Jurgens et al. 2008). These seasonal patterns in vertical hydraulic gradients suggest that shallow groundwater is driven downward in the system to a substantially greater degree during the summer than during the winter. Consequently, public-supply wells likely capture more young groundwater from the shallow part of the aquifer during summer than during winter (Figure 5a). This greater contribution of shallow groundwater, which tends to have elevated concentrations of nitrate and uranium, likely results in the commonly observed pattern of higher concentrations of nitrate and uranium in water produced by supply wells during the summer as compared to during the winter. However, the opposite seasonal pattern is observed in water quality from some Modesto wells, including the MSW (Figure 4a).

Historical data from the City of Modesto show that nitrate and uranium concentrations for the MSW are significantly higher during the winter pumping season (median nitrate 37.5 mg/L and median uranium 18.2 pCi/L) than during the summer pumping season (median nitrate 13.5 mg/L and median uranium 6.7 pCi/L; Mann-Whitney test, p < 0.001). Nitrate and uranium concentrations in samples collected from the MSW in November 2003 (winter) and August 2004 (summer) for the TANC study showed the same seasonal patterns (Jurgens et al. 2008). The MSW has a long screened interval and captures water that was recharged in recent decades near the top of the screen and water that was recharged thousands of years ago near the bottom of the screen (Jurgens et al. 2008). Groundwater sampled from the MSW in November 2003 was younger (mean age 35 years) than groundwater sampled in August 2004 (mean age 55 years), which is consistent with previous studies demonstrating that nitrate and uranium concentrations generally are higher in younger, shallower groundwater than in older, deeper groundwater (Jurgens et al. 2010).

**Figure 6:**
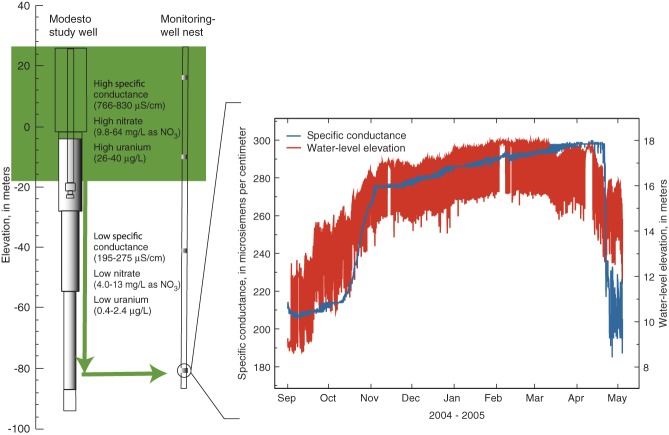
When the Modesto study well (MSW) is idle for long periods during the winter, flow through the wellbore results in movement of young groundwater with high specific conductance and elevated uranium and nitrate concentrations from shallower parts of the aquifer to greater depths, as illustrated by continuous water-level and specific-conductance data from the deep completion of a monitoring-well nest less than 30 m away (modified from Jurgens et al. 2008). When the MSW is pumped during the winter, it discharges larger amounts of contaminated groundwater than would otherwise be present if deep storage of formerly shallow groundwater did not occur.

Continuous hydraulic-head and specific-conductance data from the monitoring-well nest located within 30 m of the MSW (Figure 6) show that seasonal changes in the quality of water from the MSW are as a result of wellbore flow that occurs during prolonged periods of nonoperation. The steep vertical hydraulic gradient across the screened interval of the MSW causes shallow groundwater to enter the well or annular space and move downward through the wellbore when the well is not pumping; this flow exits the wellbore near the bottom of the well and enters the deep part of the aquifer (Jurgens et al. 2008). Movement of shallow groundwater into the deep part of the aquifer is reflected by the rise in hydraulic head and specific conductance in the deep monitoring well when the MSW has been idle for long periods (Figure 6). The head and conductance data begin to fall during the summer, when long periods of pumping cause the stored shallow groundwater to be evacuated from the deep part of the aquifer.

The quality of water from the MSW is influenced by these large volumes of shallow, contaminated groundwater flowing downward through the wellbore to be stored in deeper parts of the aquifer during the long idle periods of the MSW in the winter (Figure 5b). When the MSW is pumped during the winter, it discharges larger amounts of contaminated groundwater than would otherwise be present if deep storage of formerly shallow groundwater did not occur. The result is higher concentrations of nitrate and uranium in water produced by the MSW during the winter than during the summer. A groundwater flow model of the vicinity of the MSW (Yager and Heywood 2014) supports the conclusion that shallow groundwater flows downward through the wellbore of the MSW when it is idle.

In summary, most wells in Modesto have higher concentrations of nitrate and uranium during summer than during winter, likely caused by increased amounts of shallow groundwater reaching public-supply wells. Some deep wells with long screens have the opposite seasonal trend (Figure 4a) because strong vertical gradients in this system promote the downward movement of shallow groundwater through wellbores or annular material to the deep part of the aquifer, where it is stored until pumping resumes. For wells with shorter screened intervals, which tend to be shallower wells, weaker head gradients across the screened interval probably result in less wellbore flow during idle periods. Also, the increased contribution of shallow water reaching shallower supply wells as a result of enhanced downward gradients during the summer pumping season might substantially exceed the deep storage of any wellbore flow that occurs during winter idle periods.

### Seasonal Trends in Supply Well Water Quality in Albuquerque

Of the 95 public-supply wells in the ABCWUA historical dataset of water quality that were investigated for this study, 51 (54%) had at least one arsenic concentration above the MCL of 10 µg/L. Analysis of the historical ABCWUA dataset indicates that several individual supply wells produce groundwater with seasonal differences in arsenic concentrations, and that winter concentrations are more likely to be higher than summer concentrations. Out of the 95 public-supply wells, 17 had median arsenic concentrations below detection for both seasons; these 17 wells were excluded from further analysis because censoring precluded analysis of seasonal differences in concentration. Of the 78 wells included in further analysis of seasonal variability, 49 (63%) had seasonal differences in median arsenic concentrations of at least 1 µg/L (the resolution of the available data); 32 of these 49 wells had higher median concentrations for samples collected in the winter pumping season than in the summer pumping season. Mann-Whitney tests conducted on the 66 wells that had results for at least 10 samples for each pumping season found statistically significant differences in the seasonal distribution of arsenic concentrations in 12 wells (18%). Eleven of these 12 wells (including the ASW) had generally higher concentrations during the winter.

Examination of the ABCWUA dataset indicated that arsenic concentrations in several wells were correlated with pumping. Of the 78 wells examined, with 11 to 164 data points per well, 9 wells (12%) had positive correlations of arsenic concentrations with pumping, implying that concentrations would generally be higher during the summer, and 19 wells (24%) had negative correlations with pumping, implying that concentrations would generally be higher during the winter. Understanding the hydrologic processes that result in the observed relations of arsenic concentrations with monthly pumping was enhanced by knowledge of hydrologic and geochemical conditions at the wellbore scale, provided by investigation of the ASW by the TANC study, and at the regional scale, provided by the regional network of piezometer nests in the Albuquerque area.

On the basis of historical data from the ABCWUA (1988 to 2005), the ASW has produced raw groundwater with arsenic concentrations ranging between about 7 and 13 µg/L; therefore, the well varies between producing water that meets the drinking water standard of 10 µg/L and producing water that exceeds the standard. Arsenic concentrations typically are higher during the winter pumping season (median concentration 11 µg/L) than during the summer pumping season (median concentration 10 µg/L; Mann-Whitney test, p = 0.02), which is the most common pattern observed in the regional dataset for wells with seasonal variability. Arsenic concentrations also were higher in two winter samples (December 2007 and November 2008) than in two summer samples (June 2007 and May 2009) collected from the ASW as part of the TANC study (Bexfield et al. 2012). Groundwater produced by the ASW in the winter included a smaller fraction of young water (about 4.5%) and had older mean ages (averaging about 15,400 years) than groundwater produced in the summer, which averaged about 11% young water and about 11,300 years in mean age (Bexfield et al. 2012). This result is consistent with previous investigations demonstrating that arsenic concentrations in the area generally are higher in older, deeper groundwater (Bexfield and Plummer 2003; Plummer et al. 2004a; Bexfield et al. 2012). Similarly, VOC detections were less common in winter than in summer samples, which is consistent with a lower fraction of young groundwater in winter samples.

**Figure 7:**
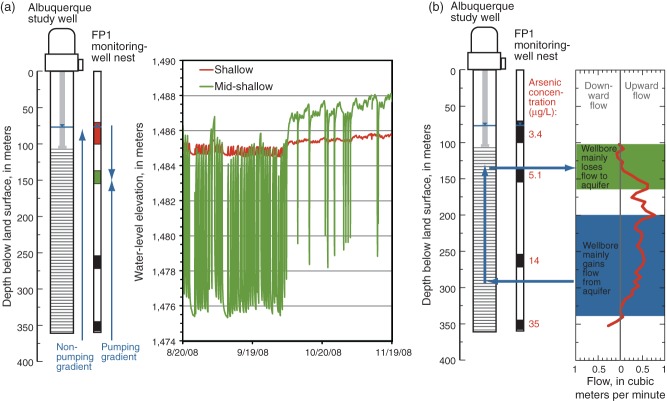
(a) Vertical hydraulic gradients near the Albuquerque supply well are upward throughout the aquifer under ambient conditions, but the gradient at shallow depths changes direction when the supply well is pumping. (b) When the supply well is not pumping, flow through the wellbore results in movement of old groundwater with high arsenic concentrations from deeper parts of the aquifer to shallower depths.

Hydraulic heads recorded in the nest of four monitoring wells located about 33 m from the ASW (Bexfield et al. 2012) indicate that vertical hydraulic gradients at that location generally are upward throughout the aquifer under ambient (non-pumping) conditions, but that the gradients change in both direction and magnitude in response to pumping of the supply well (Figure 7a). The gradient between the shallow and mid-shallow monitoring wells generally is downward when the ASW is pumping and upward when it is not; however, during the summer pumping season, the gradient can remain downward even when the ASW is off. This pattern in hydraulic gradients indicates that the generally younger, lower-arsenic groundwater in the shallow part of the aquifer near the ASW would tend to migrate farther down into the aquifer (likely making a greater contribution to water produced by the ASW) during the summer pumping season than during the winter pumping season. A groundwater flow model of the vicinity of the ASW (Yager and Heywood 2014) confirms that as a result of steeper hydraulic gradients during the summer as compared to winter, more water produced by the ASW during summer is younger groundwater entering through the upper portion of the well screen.

As also observed for the MSW, the hydraulic gradient across the screened interval of the ASW results in the movement of groundwater through the wellbore. Measurements made in the ASW during December 2007 using an electromagnetic flow meter (Bexfield et al. 2012) indicated that flow through the wellbore under ambient conditions is primarily upward and can be as much as 0.8 m^3^/min (204 gpm) at a given depth (Figure 7b). The wellbore primarily gained water from the aquifer below about 200 mbls and primarily lost water to the aquifer above about 157 mbls. Arsenic concentrations obtained from the mid-deep well (275 m deep) and the deep well (359 m deep) of the FP1 monitoring-well nest were 14 and 35 µg/L, respectively. In comparison, arsenic concentrations obtained from the shallow well (103 m deep) and mid-shallow well (158 m deep) were 3.4 and 5.1 µg/L, respectively. These patterns are consistent with wellbore flow through the ASW under ambient conditions, with old groundwater containing high arsenic concentrations moving from deeper parts of the aquifer to shallower parts of the aquifer, where it is stored and mixes with groundwater at that depth interval. Once pumping by the ASW resumes, the stored water is drawn back into the supply well, resulting in higher arsenic concentrations and higher fractions of old groundwater during the winter pumping season, when the well is often idle, as compared to during the summer pumping season (Figure 5c). A ternary mixing model designed to describe concentrations of arsenic and age tracers in samples from the ASW (Jurgens et al. 2014) found that the contribution of deep water that had been stored in shallower parts of the aquifer ranged from about 4% in one summer sample to 39% in one winter sample. The groundwater flow model of Yager and Heywood (2014) also supports the conclusion that deep groundwater flows upward through the wellbore when the ASW is idle.

Knowledge gained in the vicinity of the ASW can be expanded to other areas of Albuquerque where data on hydraulic heads and groundwater chemistry also are available. Of the 17 monitoring-well nests located across the Albuquerque area (Figure 2), arsenic data are available from Bexfield and Plummer (2003) and Plummer et al. (2004a) for 12 nests; these data indicate that arsenic generally increases with increasing depth. Head data available from Beman and Torres (2011) indicate that in parts of Albuquerque near and west of the Rio Grande, easily described as west of Interstate 25, vertical hydraulic gradients are consistently downward from the water table to the bottom of the production zone throughout the year (Figure 2). In this area, therefore, flow through the wellbore of supply wells should be downward throughout the year, from generally lower- to higher-arsenic zones of the aquifer. All nine supply wells with significant positive correlations between arsenic concentration and pumping are located in this area, which is consistent with greater downward flow of lower-arsenic water through the wellbore in the winter tending to decrease winter arsenic concentrations relative to summer concentrations (Figure 2). The magnitudes of vertical hydraulic gradients from the water table to the production zone commonly increase during the summer, potentially causing increased migration of lower-arsenic water toward the production zone. However, seasonal trends in arsenic concentrations from supply wells in this area (lower arsenic during the winter) are inconsistent with this pattern of migration of lower-arsenic water downward through the aquifer during the summer, suggesting that flow through wellbores is the more important driver of seasonal water-quality trends.

In all monitoring-well nests east of Interstate 25, vertical hydraulic gradients are upward across the entire aquifer profile, or at least between the bottom and middle of the production zone, during the winter (Figure 2). Therefore, during the winter, when decreased pumping allows for more wellbore flow, the flow should tend to be from higher- to lower-arsenic zones of the aquifer. Seventeen of the 19 supply wells with significant negative correlations between arsenic concentration and pumping are located in this area (Figure 2), which is consistent with upward flow of higher-arsenic water through the wellbore in the winter tending to increase winter arsenic concentrations relative to summer concentrations. During the summer, vertical hydraulic gradients do not change direction in some nests, but reverse from upward to downward in at least part of the aquifer in other nests. Where the gradients reverse, increased downward migration of lower-arsenic water toward the production zone would tend to enhance the pattern of lower arsenic concentrations from supply wells during that season.

### Important Aspects of Well Design and Operation in Modesto and Albuquerque

The likely causes of seasonal patterns in groundwater quality from public-supply wells in the Modesto area imply that certain changes to well design and operation might be effective in reducing the seasonal vulnerability of water from individual supply wells to contamination by nitrate and uranium. For the relatively few wells that produce water with substantially higher concentrations of nitrate and uranium during the winter than during the summer (likely more commonly deeper, longer-screened supply wells), increasing the frequency and duration of pumping during the winter could minimize the quantity of flow through the wellbore and, therefore, reduce winter concentrations of these contaminants. However, for the more common case of wells that produce water with substantially higher concentrations of nitrate and uranium during the summer than during the winter (perhaps more commonly shallower, shorter-screened supply wells), changes in operation are unlikely to reduce the summer contaminant concentrations. This is because the summer increases in nitrate and uranium concentrations are likely associated with regional patterns of groundwater pumping by multiple public-supply and irrigation wells and the increased magnitude of downward hydraulic-head gradients during the summer. Reduction of summer concentrations of nitrate and uranium in these wells would likely require changes to well completion, such as cementing off the upper part of the screen and/or deepening the well. Future wells could be designed with the causes of seasonal differences in vulnerability to selected contaminants of concern in mind.

In areas of Albuquerque where hydraulic gradients are upward during the winter, increasing the frequency and duration of pumping during the winter could minimize the quantity of high-arsenic flow through the wellbore at that time and, therefore, reduce the winter concentrations of arsenic in water produced by the well. In areas where hydraulic gradients are downward throughout the year, reducing the frequency and duration of pumping during the summer could maximize the quantity of low-arsenic flow through the wellbore at that time and, therefore, reduce the summer concentrations of arsenic in water produced by the well. In effect, an overall management strategy of generally decreased pumping in areas of downward gradients and generally increased pumping in areas of upward gradients could help optimize wellbore flow to reduce arsenic concentrations while having little effect on total withdrawals. Because data from piezometer nests indicate that arsenic concentrations generally increase with increasing depth, winter arsenic concentrations in areas of upward hydraulic gradients also could likely be reduced by cementing off the lower part of the screen and (or) designing future wells to have screened intervals that end at shallower depths. However, it is important to note that these strategies for well design and operation in the Albuquerque area increase the fraction of young water relative to old water produced by the well, thereby increasing vulnerability of water from the well to contamination with compounds related to human activities.

### Transferability to Other Alluvial Basins

The general hydrologic processes and aspects of well design that influence seasonal patterns in water quality from public-supply wells in the Modesto and Albuquerque study areas are common to alluvial basins in the southwestern United States and likely elsewhere. As in many such areas, supply wells in Modesto and Albuquerque were designed with screened intervals tens to hundreds of meters in length to maximize production. The presence of gradients in the age and quality of groundwater with depth also is common in aquifers in alluvial basins of the Southwest (Bexfield et al. 2011b). The hydrologic characteristics and processes that result in enhanced seasonal mixing of groundwater of varying depth and quality in the Modesto and Albuquerque study areas can reasonably be expected to occur in other alluvial basins with similar water-quality gradients. In particular, increased summer pumping from depths used for public supply and irrigation tends to steepen downward hydraulic gradients between the shallow aquifer and the depths of pumping. As found among many Modesto supply wells and for the ASW, seasonal increases in the magnitude of downward hydraulic gradients can be sufficient to alter the fraction of shallow groundwater captured by wells (Figure 5a). Similar findings would appear to be likely in other alluvial basins with stratified water quality and seasonally variable head gradients. Another hydrologic characteristic of both the Modesto and Albuquerque study areas that also is common in other alluvial basins with either unconfined or confined aquifer conditions is the year-round presence of substantial head gradients across vertical aquifer intervals of tens to hundreds of meters. These head gradients could commonly be sufficient to drive flow of groundwater and contaminants through the wellbores of supply wells screened across such intervals when the wells are idle (Figure 5b and 5c), as observed in both Modesto and Albuquerque.

## Summary and Conclusions

This study investigated temporal variability in the quality of groundwater produced by supply wells in Modesto, California and Albuquerque, New Mexico. The results show that the vulnerability of water from supply wells to contamination can vary throughout the year, even in groundwater systems with travel times averaging hundreds or thousands of years. In Modesto, supply wells are more likely to produce younger groundwater with higher nitrate and uranium concentrations during the summer (high) pumping season than during the winter (low) pumping season. In Albuquerque, supply wells are more likely to produce older groundwater with higher arsenic concentrations during the winter pumping season than during the summer pumping season.

In both Modesto and Albuquerque, seasonal variability in contaminant concentrations is influenced by the effects of well operations on vertical hydraulic gradients in the aquifer and on flow through the wellbores of idle supply wells. The downward migration of younger groundwater through the aquifer from shallow depths to the screened intervals of supply wells is enhanced by steep vertical gradients that develop because of increased pumping during the summer. The flow of groundwater through wellbores is enhanced by decreased pumping during the winter; the direction of this wellbore flow can be either upward or downward, depending on the direction of the vertical hydraulic gradient across the screened interval of the individual supply well. In Albuquerque, groundwater flow through wellbores is the primary driver of seasonal differences in arsenic concentrations, whereas in Modesto, enhanced vertical migration of shallow groundwater to wells is the primary mechanism leading to higher concentrations of nitrate and uranium during the summer. However, the observation of both of these drivers of seasonal variability in each study area suggests that they may play an important role in many other aquifer systems that also have stratified water quality and strong vertical gradients in hydraulic head. As in Modesto and Albuquerque, the dominant driver of seasonal variability is likely to depend upon the completion characteristics of individual wells and on local hydrologic considerations. The results of this investigation imply that in alluvial basins, general knowledge of contaminant occurrence (i.e., whether an individual contaminant is associated with young, shallow groundwater or deep, old groundwater) and of vertical hydraulic gradients allows estimation of ways in which supply wells can be designed and operated to reduce vulnerability to selected contaminants of concern.
